# Radiomic Fingerprinting of the Peritumoral Edema in Brain Tumors

**DOI:** 10.3390/cancers17030478

**Published:** 2025-02-01

**Authors:** Ghasem Azemi, Antonio Di Ieva

**Affiliations:** Computational NeuroSurgery (CNS) Lab, Macquarie Medical School, Macquarie University, Sydney, NSW 2109, Australia; antonio.diieva@mq.edu.au

**Keywords:** peritumoral region, radiomic analysis, tumor-environment interactions, glioma, meningioma, metastasis

## Abstract

Background/Objectives: Tumor interactions with their surrounding environment, particularly in the case of peritumoral edema, play a significant role in tumor behavior and progression. While most studies focus on the radiomic features of the tumor core, this work investigates whether peritumoral edema exhibits distinct radiomic fingerprints specific to glioma (GLI), meningioma (MEN), and metastasis (MET). By analyzing these patterns, we aim to deepen our understanding of the tumor microenvironment’s role in tumor development and progression. Methods: Radiomic features were extracted from peritumoral edema regions in T1-weighted (T1), post-gadolinium T1-weighted (T1-c), T2-weighted (T2), and T2 Fluid-Attenuated Inversion Recovery (T2-FLAIR) sequences. Three classification tasks using those features were then conducted: differentiating between Low-Grade Glioma (LGG) and High-Grade Glioma (HGG), distinguishing GLI from MET and MEN, and examining all four tumor types, i.e., LGG, HGG, MET, and MEN, to observe how tumor-specific signatures manifest in peritumoral edema. Model performance was assessed using balanced accuracy derived from 10-fold cross-validation. Results: The radiomic fingerprints specific to tumor types were more distinct in the peritumoral regions of T1-c images compared to other modalities. The best models, utilizing all features extracted from the peritumoral regions of T1-c images, achieved balanced accuracies of 0.86, 0.81, and 0.76 for the LGG-HGG, GLI-MET-MEN, and LGG-HGG-MET-MEN tasks, respectively. Conclusions: This study demonstrates that peritumoral edema, as characterized by radiomic features extracted from MRIs, contains fingerprints specific to tumor type, providing a non-invasive approach to understanding tumor-brain interactions. The results of this study hold the potential for predicting recurrence, distinguishing progression from pseudo-progression, and assessing treatment-induced changes, particularly in gliomas.

## Simple Summary

Brain tumors impact not only the tumor core but also the surrounding brain tissue, particularly the peritumoral edema. This study investigated whether these peritumoral regions exhibit radiomic patterns unique to different tumor types, such as gliomas, meningiomas, and metastases. Our results demonstrated that the peritumoral regions contain radiomic fingerprints indicative of tumor types, highlighting their potential as a non-invasive method for characterizing tumor behavior and interactions with the surrounding brain environment. While the primary goal of this study was not to classify tumor types, the findings pave the way for several promising applications. For instance, peritumoral radiomic analysis could help predict tumor recurrence or distinguish between true tumor progression and pseudo-progression, especially in gliomas. These insights could lead to earlier intervention and improved treatment monitoring, providing valuable tools for clinicians and advancing our understanding of tumor-brain interactions.

## 1. Introduction

Radiomics involves extracting quantitative features from medical images using data-characterization algorithms. These features capture tissue characteristics not visible to the human eye, enhancing tumor analysis for improved diagnosis, prognosis, and treatment assessment, and offering unique insights into the biological behavior of tumors [1,2].

Radiomic features have demonstrated significant potential, not only in predicting diagnoses [3,4,5], but also in key clinical outcomes for brain tumors, such as survival rates [6,7], treatment efficacy [8,9], and the likelihood of recurrence across various cancer types [10]. Beyond prognosis, radiomics also enables non-invasive monitoring of disease progression and therapeutic responses, minimizing the reliance on repeated biopsies and invasive procedures [11,12]. Additionally, recent studies have highlighted the evolving role of radiomics in neuro-oncology, the potential of radiomics features as imaging biomarkers for multiple sclerosis progression, and the use of radiomics in predicting metastatic tumor types of brain metastasis [13,14].

Studies in [15,16] have shown that radiomic features from tumor habitats can predict specific biomarkers and genetic mutations in ovarian and colorectal cancers, demonstrating their value in prognosis and precision management. Also, radiomic features from both tumor mass and peritumoral edema have been used to differentiate gliosarcoma from glioblastoma [17] and to distinguish glioblastoma from low-grade gliomas [18]. Research has also explored the use of multiparametric MRI to characterize glioblastoma regions [19] and has integrated intratumoral and peritumoral features to predict glioma grade [20], highlighting radiomics’ potential in improving classification and guiding treatment. For a detailed overview of radiomic features and their applications in brain tumor analysis, including microenvironmental assessments, readers can refer to the comprehensive reviews in [8,21,22].

Peritumoral edema involves brain regions often far beyond the tumor core and provides valuable insights into the tumor microenvironment (TME) and the dynamic interactions between the brain and the tumor [23,24,25,26]. Understanding these interactions is essential for grasping the tumor’s biology and its potential impact on adjacent structures, which is key for developing targeted therapies [27,28]. Studies have demonstrated that edema often correlates with clinical symptoms such as increased intracranial pressure, headaches, and neurological deficits [29] and that its extent and characteristics may also serve as a valuable prognostic biomarker [30,31,32].

Focusing on glioma (GLI)—including low-grade (LGG) and high-grade (HGG)—meningioma (MEN), and metastasis (MET), this study aims to investigate how tumors interact with their surrounding environment and whether these interactions produce distinct radiomic signatures in the peritumoral region that are specific to the tumor type. We hypothesize that these interactions result in unique radiomic fingerprints in the surrounding brain tissue, reflecting tumor-specific effects on the peritumoral region. To explore this, radiomic features extracted from the peritumoral edema regions of MRIs are used to perform three classification tasks: LGG-HGG, GLI-MET-MEN, and LGG-HGG-MET-MEN. To ensure comprehensive analysis, we incorporate multiple MRI sequences to assess how different tumor types influence these sequences. This multifaceted approach helps identify which MRI sequence most effectively captures the tumor-specific radiomic fingerprints in the peritumoral region.

## 2. Materials and Methods

### 2.1. Materials

The dataset utilized for this study was the Brain Tumor Segmentation (BraTS) 2023 challenge training dataset, available at [33]. It includes 2416 subjects across three tumor types: MET (165 cases), GLI (1251 cases including 325 HGG and 77 LGG), and MEN (1000 cases). Note that, for the GLI subset, only a portion of the cases (325 HGG and 77 LGG) have available labels. Each subject’s dataset includes four MRI sequences: T1-weighted (T1), post-gadolinium T1-weighted (T1-c), T2-weighted (T2), and T2 Fluid Attenuated Inversion Recovery (T2-FLAIR), which were all used for analysis in this study, alongside a brain tumor segmentation mask outlining tumor regions.

**Figure 1:**
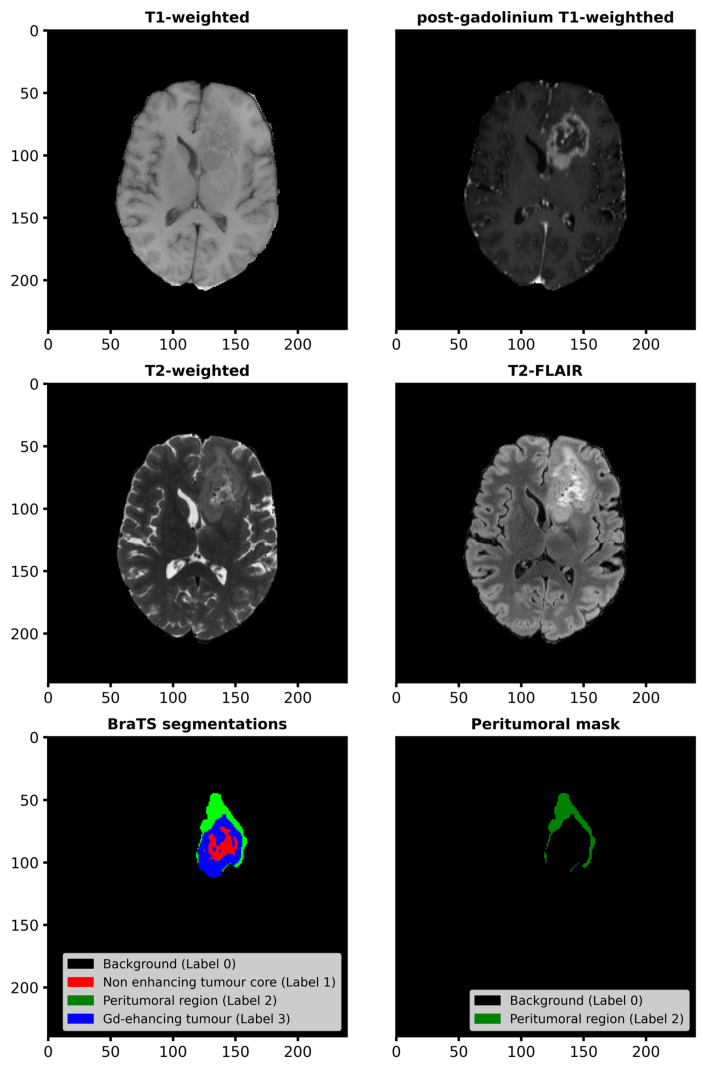
The images display slices from the T1-weighted and post-gadolinium T1-weighted sequences (**top row**), T2-weighted and T2 Fluid Attenuated Inversion Recovery sequences (**middle row**), along with the tumor segmentation (**third row, left**) and the extracted peritumoral region mask (i.e., edema - **third row, right**), used for radiomics analysis. Data are from subject BraTS-GLI-00000-000 in the BraTS 2023 challenge training set.

For each subject, the annotated tumor sub-regions include the Gd-enhancing tumor (label 3), peritumoral edematous/infiltrated tissue (label 2), and non-enhancing tumor core (label 1). Figure 1 shows a slice from the T1, T1-c, T2, and T2-FLAIR sequences of subject BraTS-GLI-00000-000 from the dataset, along with its original segmentations and the mask delineating the peritumoral region (representing edematous and/or infiltrated tissue) extracted from it. This mask was used in this study to identify the region of interest for extracting radiomic features.

To explore potential tumor-specific patterns, we focused on radiomic features extracted from the edematous peritumoral region. For this purpose, we included only subjects with Label 2 in the BraTS segmentations. This approach allows for the exploration of potential tumor-specific peritumoral radiomics fingerprinting. Subjects with issues in feature extraction from any of the four MRI sequences, such as NaN values or unsuccessful extractions, were excluded. A summary of subject counts by tumor type, including the total number of subjects, those without segmentations, those lacking the Label 2 region, those with feature extraction issues, and the final number of subjects used in this study, is provided in Table 1.

### 2.2. Methods

#### 2.2.1. Workflow for Radiomic Feature Extraction and Tumor Classification

**Figure 2:**
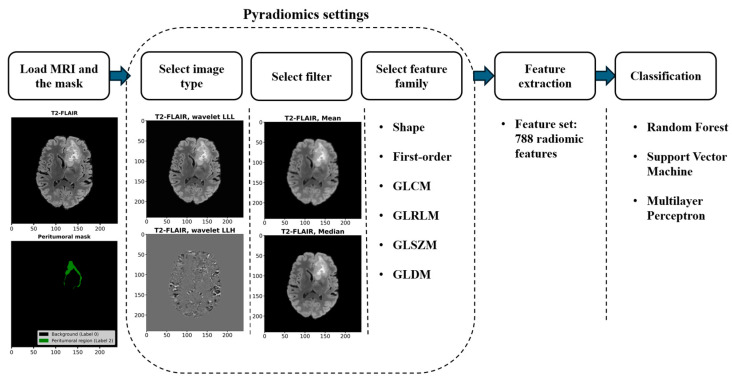
Workflow for extracting and classifying radiomic features from MRI scans. MRIs and masks from the BraTS 2023 training dataset were used. PyRadiomics extracted features from original and filtered images, which were classified using three different classifiers. The figure shows sample inputs and examples of wavelet sub-band and filtered images. Abbreviations: GLCM: Gray Level Co-occurrence Matrix, GLRLM: Gray Level Run Length Matrix, GLSZM: Gray Level Size Zone Matrix, and GLDM: Gray Level Dependence Matrix.

Figure 2 shows the workflow used for analyzing and classifying MRIs using radiomic features from peritumoral regions. Data from the BraTS 2023 training dataset included pre-processed and segmented images. Radiomic features were extracted from the peritumoral regions of MRIs using PyRadiomics, an open-source Python library designed for standardized feature extraction [34]. The extracted features were then resampled for class imbalance and selected for relevance. These features trained machine learning models to classify tumor types based on radiomic signatures. All analysis was done in Python 3.11.3 via Anaconda (April 2023).

#### 2.2.2. Radiomic Feature Extraction

PyRadiomics facilitates the extraction of a comprehensive set of features from regions of interest in medical images, enabling detailed quantitative analysis. The features include 14 shape features from the mask and 86 image-based features composed of: 18 first-order statistics, 22 gray level co-occurrence matrices (GLCM), 16 gray level run length matrices (GLRLM), 16 gray level size zone matrices (GLSZM), and 14 gray level dependence matrices (GLDM) features. Comprehensive details on all radiomic features extracted using PyRadiomics, including the mathematical formulas used for their derivation, can be found in [34], and PyRadiomics documentation is available at: https://pyradiomics.readthedocs.io/en/latest/ (accessed on 29 January 2025).

This study used PyRadiomics to extract a radiomic feature set capturing different aspects of the MRI data through varied image transformations and filtering techniques. This set contained 14 shape features from the mask and 9 × 86 features extracted from the original image, wavelet sub-band images, and Laplacian of Gaussian (LoG) filtered images. The wavelet transforms encompassed all combinations of high and low pass filters, while LoG filters were applied with sigma values of 2, 3, 4, and 5. Consequently, the feature set included 788 radiomic features. Examples of wavelet sub-band and filtered images from a representative MRI scan are shown in Figure 2.

It is important to note that the dataset includes cases with multiple lesions. However, in many instances, the smaller lesions comprise less than 1% of the total voxels in the scan. PyRadiomics allows for the extraction of features from non-contiguous regions within the segmentation mask, treating multiple separated regions as a single region of interest (ROI) for feature extraction. Feature calculations are performed solely on voxels marked as True in the mask, irrespective of their spatial location. While this approach may influence the calculation of certain features, especially shape-related ones, the primary focus of this study is on the discriminatory power of radiomic features for tumor classification.

#### 2.2.3. Feature Normalization, Data Imbalance Handling, and Feature Selection

All features were normalized using MinMax scaling, which transforms feature values to a range between 0 and 1. This step ensures that the magnitude of each feature does not disproportionately affect the analysis. Moreover, to address data imbalance, Random Under Sampling (RUS) was employed to reduce the majority class, achieving a more balanced dataset. RUS is a commonly used technique for tackling class imbalance in machine learning tasks, as it helps prevent the model from being biased toward the majority class. Additionally, the Synthetic Minority Over-sampling Technique (SMOTE) can also be utilized to address class imbalance by generating synthetic samples for the minority class, thereby enhancing the model’s ability to learn from underrepresented data. However, since the focus of this study was to investigate tumor-specific fingerprints of radiomic features extracted from edema regions and considering that SMOTE-balancing resulted in lower performance compared to RUS for the LGG-HGG classification task, we opted to use RUS as the preferred data balancing technique for faster training.

Finally, for feature selection, Principal Component Analysis (PCA) was applied to reduce the dimensionality of the feature space while retaining the most significant variance in the data. PCA is a widely used method for transforming high-dimensional data into a lower-dimensional form, ensuring that the most important features are preserved for subsequent analysis.

#### 2.2.4. Classification and Performance Evaluation

We analyzed three tumor differentiation tasks—LGG-HGG, GLI-MET-MEN, and LGG-HGG-MET-MEN—using radiomic features extracted from peritumoral regions. To evaluate these features, we employed three classifiers: Random Forest (RF), Multilayer Perceptron (MLP), and Support Vector Machine (SVM). The classifiers’ hyperparameter distributions were specified using Python’s scipy.stats and sklearn libraries (as detailed in Table 2).

A 10-fold cross-validation approach was used to ensure reliability, with each subset serving as the validation set once while the others were used for training. Radiomic features were analyzed across T1, T1-c, T2, and T2-FLAIR MRI sequences, with 10 iterations performed over the hyperparameter distributions to identify the best-performing models. Within each fold, classifier parameters, along with the number of PCA, were optimized using the training data only. RUS was applied to address class imbalance, and two feature selection methods—None and PCA—were tested. Confusion matrices were generated to compute key performance metrics, including balanced accuracy, sensitivity, specificity, and F1-score. The best setting was determined by achieving the highest balanced accuracy.

#### 2.2.5. SHAP Analysis for Feature Interpretability

To enhance the interpretability and feature selection of our brain tumor classification model, we employed SHAP (SHapley Additive exPlanations) values to identify the most influential radiomic features extracted from MRI scans [35]. SHAP values provide a consistent and objective explanation of how each feature impacts the model’s predictions, allowing us to quantify the contribution of individual radiomic features to the classification outcome.

We implemented our classification task using 10-fold cross-validation to ensure robust performance evaluation. For each fold, we calculated SHAP values for all extracted radiomic features and ranked them based on their absolute SHAP values, which represent the magnitude of their impact on the model’s predictions. We then selected the top 5 features with the highest absolute SHAP values for each fold, resulting in a total of 50 features across all 10 folds. Finally, to determine the most consistently important features across all folds, we analyzed the frequency of occurrence of these 50 features. The 10 features that appeared most frequently in this list of 50 were identified as our final set of top contributive features for the brain tumor classification task.

This SHAP-based feature selection approach, combined with cross-validation, not only helps in identifying the most relevant and consistent radiomic markers for brain tumor classification but also provides insights into the model’s decision-making process, enhancing the overall interpretability and reliability of our classification pipeline.

## 3. Results

The findings from investigating tumor-specific radiomic fingerprints in the peritumoral regions, through the three tumor-type classification tasks, are detailed in this section.

### 3.1. Results for the LGG-HGG Classification Task

Table 3 summarizes the performance of radiomic features from peritumoral regions in distinguishing LGG from HGG across T1, T1-c, T2, and T2-FLAIR imaging modalities. It reports the balanced accuracy for each combination of imaging modality and feature selection approach. Among the modalities, radiomic features derived from T1-c images demonstrated the strongest differentiation, with the top model achieving a balanced accuracy of 0.86 using RUS to overcome data imbalance without applying feature selection. Features extracted from T1 images ranked second, achieving a balanced accuracy of 0.74 under the same configuration of RUS and no feature selection.

**Figure 3:**
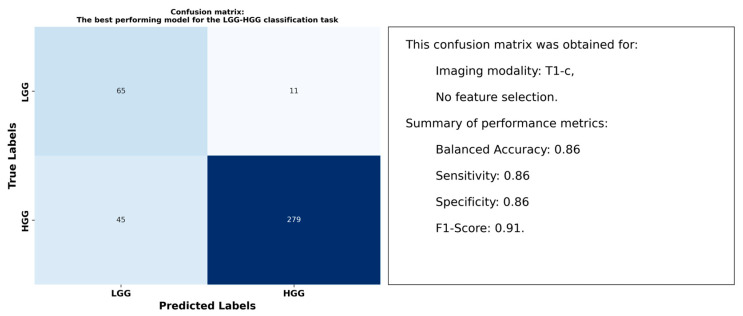
Confusion matrix for the top model in distinguishing between LGG and HGG tumor types based on radiomic features extracted from peritumoral regions.

Figure 3 displays the confusion matrix for the model with the highest balanced accuracy of 0.86. It includes details on the imaging modality, along with key performance metrics for a comprehensive view of the model’s classification capabilities.

**Figure 4:**
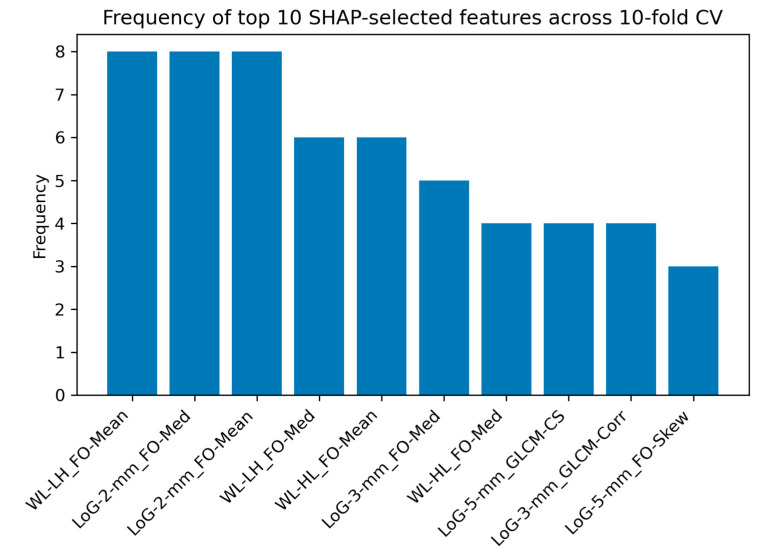
Frequency of the top 10 SHAP-selected features across the 10-fold cross-validation for the LGG-HGG classification task, highlighting the most influential radiomic markers. Abbreviations: WL: Wavelet, FO: First-Order, LoG: Laplacian of Gaussian, Med: Median, GLCM: Gray Level Co-occurrence Matrix, CS: Cluster Shade, Corr: Correlation, and Skew: Skewness.

Figure 4 shows the frequency of the top 10 SHAP-selected features across the 10-fold cross-validation for the LGG-HGG classification task. These features were identified based on their influence on the model’s predictions, with the frequency reflecting their consistency in contributing to the classification outcome. The figure provides valuable insights into the most important radiomic features for distinguishing between the LGG and HGG cases.

### 3.2. Results for the GLI-MEN-MET Classification Task

Table 4 highlights the ability of radiomic features from peritumoral regions to distinguish between GLI, MET, and MEN tumor types using data from T1, T1-c, T2, and T2-FLAIR imaging modalities. The table presents balanced accuracy for each combination of imaging modality and feature selection method. T1-c images demonstrated the strongest differentiation, achieving the highest balanced accuracy of 0.81. This result was obtained using RUS for data balancing, with no additional feature selection applied. Features extracted from FLAIR images followed closely, achieving the second-best balanced accuracy of 0.79 under the same configuration of RUS and no feature selection.

**Figure 5:**
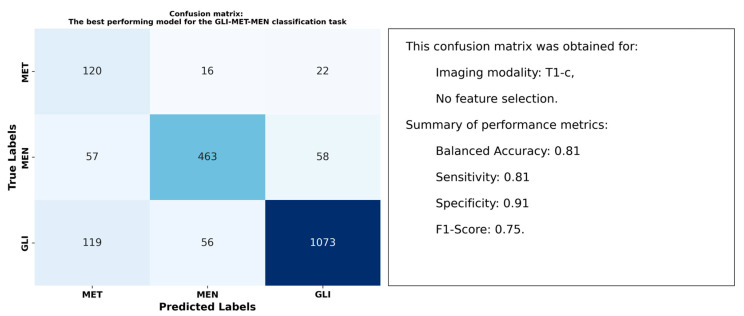
Confusion matrix illustrating the performance of the top model in distinguishing between GLI, MET, and MEN tumor types.

Figure 5 illustrates the confusion matrix for the highest-performing model, which achieved a balanced accuracy of 0.81 for this classification task. It includes key performance metrics to provide a thorough assessment of the model’s effectiveness.

**Figure 6:**
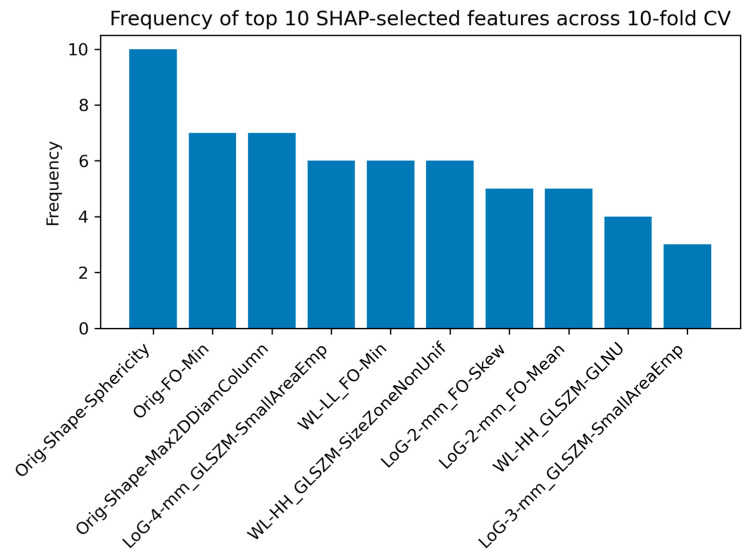
Frequency of the top 10 SHAP-selected features across the 10-fold cross-validation for the LGG-HGG classification task, highlighting the most influential radiomic markers. Abbreviations: Orig: Original, FO: First-Order, Min: Minimum, Max: Maximum, Diam = Diameter, LoG: Laplacian of Gaussian, GLSZM: Gray Level Size Zone Matrix, WL: Wavelet, Skew: Skewness, GLNU: Gray Level Non-Uniformity, and SmallAreaEmp: Small Area Emphasis.

Figure 6 presents the frequency of the top 10 SHAP-selected features across the 10-fold cross-validation for the GLI-MEN-MET classification task. These features represent the most consistent contributors to the model’s predictions, providing insight into the key radiomic markers distinguishing GLI, MEN, and MET.

### 3.3. Results for the LGG-HGG-MET-MEN Classification Task

Table 5 highlights the performance of radiomic features from peritumoral regions in differentiating between HGG, LGG, MET, and MEN tumor types across T1, T1-c, T2, and T2-FLAIR imaging modalities. The highest balanced accuracy of 0.76 was achieved using T1-c features, with RUS applied for data balancing and no feature selection. T2 images ranked second, yielding a balanced accuracy of 0.67 under the same configuration.

**Figure 7:**
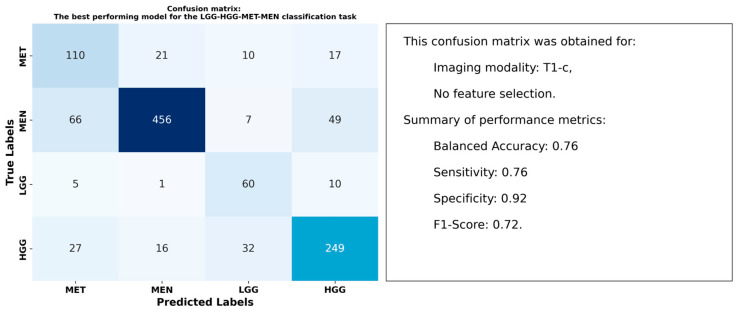
Confusion matrix demonstrating the performance of the best model in differentiating between LGG, HGG, MET, and MEN tumor types using peritumoral radiomic features.

Figure 7 presents the confusion matrix for the model that achieved the highest balanced accuracy of 0.76 in the LGG-HGG-MET-MEN classification task. Along with the confusion matrix, key performance metrics are included to offer a comprehensive evaluation of the model’s ability to differentiate between tumor types.

**Figure 8:**
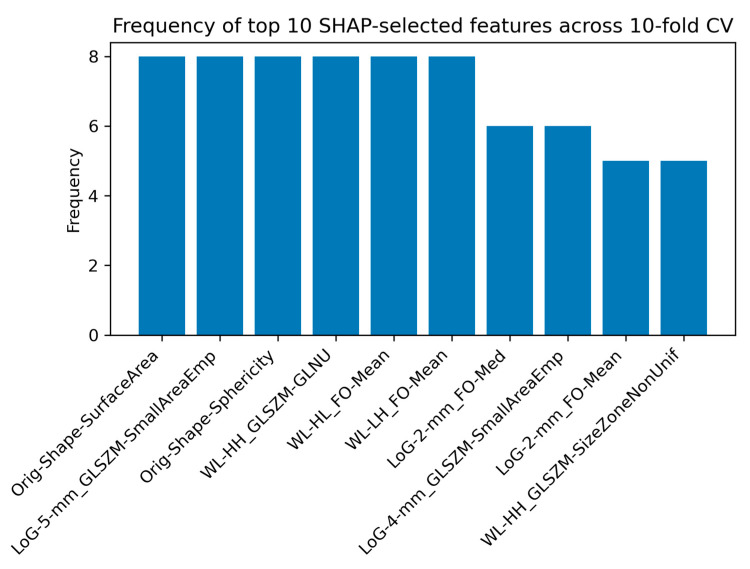
Frequency of the top 10 SHAP-selected features across the 10-fold cross-validation for the LGG-HGG-MET-MEN classification task, emphasizing the most influential radiomic features. Abbreviations: Orig = Original, LoG = Laplacian of Gaussian, WL = Wavelet, FO = First-Order, GLSZM = Gray Level Size Zone Matrix, GLNU = Gray Level Non-Uniformity, SmallAreaEmp = Small Area Emphasis, Med = Median.

Figure 8 illustrates the frequency of the top 10 SHAP-selected features across the 10-fold cross-validation for the LGG-HGG-MET-MEN classification task. These features highlight the most consistent contributors to the model’s predictions, offering valuable insights into the key radiomic markers differentiating LGG, HGG, MET, and MEN.

## 4. Discussion

### 4.1. Distinct Radiomic Signatures of Peritumoral Edema

In this study, we investigated how brain tumors affect their surrounding peritumoral edema to identify distinct tumor-specific patterns. Instead of focusing on direct tumor classification, we explored whether the peritumoral regions have unique signatures that could indicate tumor types. We extracted radiomic features from peritumoral regions in T1, T1-c, T2, and T2-FLAIR MRI images, focusing on tumor types such as LGG, HGG, MET, and MEN. These patterns were assessed through three classification tasks: distinguishing the peritumoral edematous regions between LGG and HGG, among GLI, MET, and MEN, and across all four tumor types—LGG, HGG, MET, and MEN. Including meningiomas in this study was essential for exploring how different tumors affect the surrounding brain tissue. Although differentiating gliomas from meningiomas may seem a trivial exercise, their inclusion allowed us to assess whether peritumoral edema exhibits radiomic patterns that are uniquely tied to the underlying tumor type (i.e., infiltrative and diffuse cancer, such as glioma, vs. a compressive tumor, such as meningioma grade 1). This approach deepens our understanding of tumor-environment interactions and highlights whether radiomic signatures can capture the distinct biological characteristics of gliomas, metastases, and meningiomas alike.

Our results demonstrated that peritumoral regions do exhibit radiomic fingerprints that are indicative of tumor types. This highlights the potential of peritumoral radiomic analysis as a non-invasive approach for characterizing tumor behavior and interaction with surrounding brain tissue. It is important to clarify that the primary aim of this study was not tumor type classification. Had classification been the main objective, radiomic features from the tumor core would have been included, as prior research, e.g., [20,36], has demonstrated that incorporating such features can significantly improve classification accuracy. We, therefore, expect that applying the methodology presented in this study to the tumor core would likely result in higher accuracies compared to those achieved using radiomic features from the edema regions.

### 4.2. Optimal Models and Superior Performance of T1-c Features

Our results indicated that models using radiomic features from T1-c images consistently outperformed those using other images. For the three classification tasks performed, T1-c features achieved balanced accuracies of 0.86 for LGG-HGG, 0.81 for GLI-MET-MEN, and 0.76 for the more complex LGG-HGG-MET-MEN task. These results emphasize the superior performance of T1-c features in brain tumor classification, even when focusing solely on peritumoral regions.

The enhanced performance of T1-c features is attributed to their superior contrast, which improves visualization of blood-brain barrier disruption—a common tumor characteristic. This is likely to contribute to more accurate feature extraction. Previous studies also support the effectiveness of T1-c features in tumor classification [37,38]. The better performance of T1-c radiomic features suggests that they are more effective at capturing tumor-specific characteristics compared to features from other sequences, highlighting their specificity to tumor types. While T2-FLAIR remains crucial for accurately delineating tumor boundaries and identifying peritumoral edema—especially in low-grade gliomas and for better defining peritumoral regions in any brain tumor—it is the features extracted from T1-c images that provide more precise information related to tumor type, underscoring the complementary roles of these sequences in radiomic analysis.

### 4.3. Impact of Data Balancing and Feature Selection Techniques

Our findings indicated that RUS, which balances the dataset by removing samples from the majority class, performed well and consistently contributed to better classification results. We also evaluated SMOTE as a data balancing technique when using all radiomic features extracted from the edema regions of T1-c scans across all three classification tasks. Our findings showed that, for the LGG-HGG classification task, RUS achieved a balanced accuracy of 86%, whereas SMOTE resulted in 82%, for the GLI-MEN-MET task, RUS achieved 82% while SMOTE resulted in 83%, and, for the LGG-HGG-MET-MEN task, RUS yielded 78% compared to 73% with SMOTE. While SMOTE works by generating synthetic samples for the minority class, this approach can sometimes introduce noise and lead to overfitting, as the synthetic data may not fully capture the true variability of the radiomic features in peritumoral regions.

When comparing the settings using all features to those employing feature selection methods, we observed that settings using all features achieved slightly better performance in the three classification tasks. However, settings with PCA-selected features also performed well, indicating that PCA efficiently reduced dimensionality while retaining key features. These findings are consistent with a review in [21] on radiomics and machine learning in brain tumors, which emphasized the importance of optimizing radiomic feature extraction and standardizing imaging protocols for improved classification accuracy.

### 4.4. Interpretation of Radiomic Features Using SHAP Analysis

SHAP analysis was conducted to identify the most influential radiomic features contributing to the classification of brain tumors across different tasks. Figure 4, Figure 6 and Figure 8 present the frequency of the top 10 SHAP-selected features across the 10-fold cross-validation process for each classification task.

For the LGG-HGG classification task, the three most frequently selected features were Wavelet-LH First-Order Mean, LoG with sigma 2 mm First-Order Median, and LoG with sigma 2 mm First-Order Mean, each appearing in 8 out of 10 folds. These first-order statistical features, derived from wavelet-transformed and LoG-filtered images, highlight the importance of intensity-based characteristics in differentiating LGG and HGG. Their consistent selection suggests that variations in intensity distribution within the peritumoral edema region provide critical information for tumor grading.

In the GLI-MEN-MET classification task, the most consistently selected features were Original Shape Sphericity, appearing in all 10 folds, followed by Original First-Order Minimum and Original Shape Maximum 2D Diameter Column, both selected in 7 out of 10 folds. These features, reflecting tumor shape and first-order intensity characteristics from unfiltered images, suggest that intrinsic morphological properties and intensity variations play a key role in GLI, MEN, and MET. The recurrence of these features highlights the importance of structural attributes in tumor classification.

For the LGG-HGG-MET-MEN classification task, the most frequently selected features were Original Shape Surface Area, LoG with 5 mm sigma GLSZM Small Area Emphasis, and Original Shape Sphericity, each appearing in 8 out of 10 folds. These features capture a combination of morphological complexity and texture variations, emphasizing the significance of geometric properties and fine-grained intensity patterns within the edema region in distinguishing the four tumor types.

Overall, the consistent selection of specific radiomic features across multiple folds underscores their robustness and relevance in tumor classification, reinforcing the potential of radiomic analysis in capturing meaningful tumor characteristics across different tumor types.

### 4.5. Future Directions in Peritumoral Radiomics and Clinical Applications

While this study primarily focused on exploring the edematous peritumoral radiomic patterns for tumor differentiation, it opens several promising avenues for future research and clinical application. One significant application is the potential for characterizing peritumoral fingerprinting to predict tumor recurrence—a critical challenge in oncology. By identifying unique radiomic patterns in the peritumoral regions, we may be able to predict tumor recurrence or progression, allowing earlier intervention, especially in cases of gliomas and metastases [39,40]. Another vital area is distinguishing between tumor progression and pseudo-progression, particularly in gliomas, where differentiating between actual tumor growth and treatment-related changes can be difficult.

In addition, a promising direction for future research is understanding how the brain reacts to non-surgical treatments such as radiotherapy and novel immunotherapy strategies. These treatments can significantly alter the peritumoral environment, and the ability to monitor these changes through radiomic features could provide a deeper understanding of treatment efficacy and the underlying biological processes [41]. Developing robust models of brain radiomics may pave the way for new insights into cancer treatment and management. Although these applications are beyond the scope of this study, they represent exciting future research opportunities.

Future research will also aim to expand the applicability of peritumoral radiomics to other tumor types and subtypes (e.g., meningioma grade 1 vs. 2, considering that the latter can infiltrate surrounding brain parenchyma) and stages to assess their generalizability and robustness [42]. This includes exploring radiomic features from the brain hemisphere where the tumor predominantly resides, excluding both Gd-enhancing and non-enhancing tumor cores, to reveal additional insights into tumor-environment interactions. Another critical direction will be assessing how treatments such as anti-angiogenic drugs affecting the peritumoral edema, such as bevacizumab, or immunotherapy, influence the peritumoral region [43]. Furthermore, expanding radiomic and radiogenomic analyses to correlate these quantitative maps to other -omics approaches (e.g., genomics, transcriptomics, and proteomics of tumors and their surrounding regions) is a further exciting area of future investigations [44].

### 4.6. Limitations

This study is constrained by the relatively small and homogeneous dataset, which may limit the generalizability of the findings on tumor-environment interactions. Expanding the dataset to include a broader range of cases (including different tumor subtypes) and populations would help validate these results and enhance their applicability. Additionally, incorporating other imaging techniques, such as diffusion-weighted imaging (DWI) or magnetic resonance spectroscopy (MRS), could provide additional insights into the tumor-environment relationship and potentially reveal more nuanced interactions.

## 5. Conclusions

This study’s unique focus on radiomic features extracted from the tumor’s surrounding edema, rather than the tumor itself, provides valuable insights into tumor classification and its interactions with surrounding tissues. By analyzing these regions, we successfully differentiated between various tumor types and highlighted the influence of tumors on their environments, suggesting that peritumoral edema harbors distinct pathological signatures. Specifically, we demonstrated that the edema associated with high- and low-grade gliomas, meningiomas, and metastases exhibits radiomic fingerprints unique to each tumor type, emphasizing the diagnostic potential of peritumoral regions. The findings further reinforce the relevance of peritumoral edema in tumor characterization, showcasing its ability to provide complementary information to traditional tumor-focused analyses. This non-invasive approach enhances our understanding of tumor-brain interactions and underscores the role of the tumor microenvironment in disease progression.

The results of this study highlight the potential of integrating radiomic analysis into clinical workflows, contributing to improved patient stratification, treatment planning, and decision-making. By utilizing the radiomic signatures of peritumoral edema, this study paves the way for advancements in personalized medicine and the non-invasive management of brain tumors.

## Figures and Tables

**Table 1:** Summary of subjects counts across different categories. This table lists the total number of subjects, the number without segmentations, those lacking the Label 2 region, those with feature extraction issues, and the number of subjects ultimately used in this study. Abbreviations: MET: Metastasis, GLI: Glioma, HGG: High-Grade Glioma, LGG: Low-Grade Glioma, and MEN: Meningioma.

Group	Total Subjects	Excluded Subjects			Subjects in Study
		No Segmentations	No Label 2	Feature Extraction Issues	
MET	165	0	4	3	158
GLI	1251	0	1	2	1248
HGG	325	0	0	1	324
LGG	77	0	1	0	76
MEN	1000	22	335	65	578
Total	2818	22	341	71	2384

**Table 2:** Hyperparameter distributions for the classifiers used in the study, specified with Python’s scipy.stats and sklearn libraries. The table details parameters, and their ranges or distributions, for the three classifiers used in this study. Abbreviations: RF: Random Forest, MLP: Multi-Layer Perceptron, and SVM: Support Vector Machine.

Classifier	Parameter	Distribution/Value
RF	n_estimators	randint(100, 500)
	max_depth	[None] + list(randint(10, 50).rvs(size = 4))
	criterion	[‘entropy’, ‘gini’, ‘log_loss’]
	min_samples_split	[2, 3, 4, 5]
	max_features	[‘sqrt’, ‘log2’, None, 0.5, 0.7]
MLP	hidden_layer_sizes	[(5, 3), (5,), (5, 5), (10,), (10, 10)]
	activation	[‘relu’, ‘logistic’, ‘tanh]
	alpha	uniform(0.0001, 0.1)
	solver	[‘adam’, ‘sgd’]
SVM	C	uniform(0.1, 100)
	gamma	[‘scale’, ‘auto’, 0.1, 0.01]
	kernel	[‘linear’, ‘rbf’, ‘poly’, ‘sigmoid’]
	degree	randint(2, 5)
	coef0	uniform(0.0, 10.0)

**Table 3:** Summary of classification performance in differentiating LGG from HGG using radiomic features from peritumoral regions: The highest-performing model attained a balanced accuracy of 0.86 with features extracted from T1-c images, utilizing RUS for data balancing without applying feature selection. Abbreviation: PCA: Principal Component Analysis.

Imaging Modality	Feature Selection Method	Balanced Accuracy
T1	None	0.74
	PCA	0.67
T1-c	None	0.86
	PCA	0.83
T2	None	0.71
	PCA	0.70
T2-FLAIR	None	0.65
	PCA	0.62

**Table 4:** Overview of classification results for distinguishing GLI, MET, and MEN using radiomic features from peritumoral regions: The best model achieved a balanced accuracy of 0.81 using T1-c radiomic features. This performance was obtained with RUS applied for data balancing and no feature selection. Abbreviation: PCA: Principal Component Analysis.

Imaging Modality	Feature Selection Method	Balanced Accuracy
T1	None	0.74
	PCA	0.75
T1-c	None	0.81
	PCA	0.80
T2	None	0.76
	PCA	0.76
T2-FLAIR	None	0.79
	PCA	0.77

**Table 5:** Summary of classification results for differentiating HGG, LGG, MET, and MEN using radiomic features from the peritumoral region: The highest-performing model achieved a balanced accuracy of 0.76 with T1-c radiomic features, utilizing RUS for data balancing and no feature selection. Abbreviation: PCA = Principal Component Analysis.

Imaging Modality	Feature Selection Method	Balanced Accuracy
T1	None	0.65
	PCA	0.60
T1-c	None	0.76
	PCA	0.73
T2	None	0.67
	PCA	0.64
T2-FLAIR	None	0.63
	PCA	0.62

## Data Availability

The original data presented in the study are openly available on Kaggle at https://www.kaggle.com/datasets/bkb2024/brats-2023-training (accessed on 29 January 2025).

## Abbreviations

The following abbreviations are used in this manuscript:

DTI	Diffusion tensor imaging
FLAIR	Fluid-attenuated inversion recovery
FO	First-order
GLCM	Gray-level co-occurrence matrix
GLDM	Gray-level dependence matrix
GLI	Glioma
GLRLM	Gray-level run length matrix
GLSZM	Gray-level size zone matrix
HGG	High-grade glioma
LGG	Low-grade glioma
LoG	Laplacian of Gaussian
MEN	Meningioma
MET	Metastasis
MLP	Multilayer perceptron
MRI	Magnetic resonance imaging
MRS	Magnetic resonance spectroscopy
NaN	Not a number
PCA	Principal component analysis
RF	Random forest
ROI	Region of interest
RUS	Random under sampling
SMOTE	Synthetic minority over-sampling technique
SVM	Support vector machine
TME	Tumor microenvironment
WL	Wavelet
